# Dataset of monthly downscaled future vapor pressure projections for the conterminous USA for RCP 4.5 and RCP 8.5 compatible with NEX-DCP30

**DOI:** 10.1016/j.dib.2023.109169

**Published:** 2023-04-20

**Authors:** Raymond J. Drapek, Bridget L. Thrasher, John B. Kim

**Affiliations:** a USDA Forest Service Western Wildland Environmental Threat Assessment Center, 3200 SW Jefferson Way, Corvallis, OR 97331, USA; b Climate Analytics Group, Boulder, CO, 80305, USA

**Keywords:** NEX-DCP30, Bias correction spatial disaggregation, BCSD, PRISM, Vapor pressure, Statistical downscaling, humidity, Climate change

## Abstract

Models that simulate ecosystems at local to regional scales require relatively fine resolution climate data. Many methods exist that downscale the native resolution output from global climate models (GCM) to finer resolutions. NASA NEX-DCP30 is a statistically downscaled 30 arcsecond resolution climate dataset widely used for climate change impact studies in the conterminous USA (CONUS), but it did not include vapor pressure data which is essential for many types of models. We downscaled vapor pressure data from 28 global climate models included in the Coupled Model Intercomparison Project Phase 5 (CMIP5) to 30 arcsecond resolution for CONUS to augment the NEX-DCP30 dataset. Monthly vapor pressure values were calculated from raw GCM output for the conterminous USA from 1950 to 2100, representing RCP4.5 and RCP8.5 climate change scenarios. Vapor pressure data were then downscaled from the GCM's native spatial resolutions to 30 arcsecond using the Bias Correction-Spatial Disaggregation (BCSD) statistical downscaling method, which had been used to create the original NEX-DCP30 dataset. PRISM LT71m gridded climate data for 1970-1999 served as the reference data. The newly created downscaled vapor pressure dataset may be used in conjunction with the existing NEX-DCP30 data as input for vegetation, fire, drought, or earth system models. The data is available at the Forest Service Research Data Archive.

**Specifications Table**

**Table:** 

Subject	Climatology
Specific subject area	Statistical downscaling of climate data from global climate models.
Type of data	Spatial data
How the data were acquired	Bias Correction-Spatial Disaggregation (BCSD) statistical downscaling was applied to global climate model output.
Data format	Raw, netCDF format
Description of data collection	Monthly vapor pressure data covering the conterminous USA at 30 arcsecond spatial resolution from 1950 to 2099 or 2100 for 28 global climate models included in the Coupled Model Intercomparison Project Phase 5, representing RCP4.5 and RCP8.5 climate change scenarios
Data source location	Coupled Model Intercomparison Project 5 (CMIP5) data portal at the Lawrence Livermore National Laboratory (LLNL) node of the Earth System Grid Federation (ESGF), Berkeley, CA USA. https://esgf-node.llnl.gov/search/cmip5/
Data accessibility	Repository name: Forest Service Research Data Archive Data identification number: 10.2737/RDS-2023-0001 Direct URL to data: https://www.fs.usda.gov/rds/archive/catalog/RDS-2023-0001
Related research article	Srivastava, L., Hand, M., Kim, J., Sánchez, J.J., Lupi, F., Garnache, C., Drapek, R.J. and Quinn, J.F., 2020. How will climate change affect the provision and value of water from public lands in Southern California through the 21st century? *Agricultural and Resource Economics Review*, *49*(1), pp.117-149. https://doi.org/10.1017/age.2020.3

## Value of the Data

• Vapor pressure data is necessary input for many vegetation, fire, drought, or earth system models.
• This dataset is relatively high resolution at 30 arcseconds, approximately 800m.
• Covers the conterminous USA (“lower 48 states”) for RCP4.5 and RCP8.5 climate change scenarios from 1950 to 2100.
• Augments and is compatible with NASA NEX-DCP30 downscaled climate projections dataset.

## 1 Objective

Vapor pressure is the amount of water vapor held in the air, and is used in simulations that support climate change impact studies [1], including vegetation modeling [2,3], and wildfire modeling [4,5]. Spatially explicit vegetation or fire models require vapor pressure dataset in a gridded format, along with other climate variables. When such models are applied at local or regional scales to explore climate change impacts, they require climate data at a finer resolution than the native global climate model (GCM) resolution. NASA NEX-DCP30 is a dataset comprising gridded future climate projections covering the conterminous USA from 1950 to 2100 at a monthly time step at 30 arcsecond resolution [6]. NEX-DCP30 was created by applying the Bias Correction Spatial Disaggregation method [7] to GCM output. The original NEX-DCP30 dataset includes climate projections from 33 GCMs published by Couple Model Intercomparison Project Phase 5 (CMIP5; [8]) and for two RCP scenarios [9], but does not include vapor pressure. We downscaled vapor pressure data for 28 of the 33 GCMs in the NEX-DCP30 dataset, so that NEX-DCP30 dataset may be used to drive a vegetation model that requires vapor pressure data.

## 2 Data Description

The dataset described herein represents vapor pressure at a spatial and temporal resolutions and extents identical to NASA NEX-DCP30. It includes 28 of the 33 GCMs included in the original NASA NEX-DCP30 dataset (Table 1). It represents vapor pressure for the conterminous USA at a 30 arcsecond spatial resolution (approximately 800m at this latitude). The values are given at a monthly time step, from 1950 to 2099 or 2100, for each of the 28 GCMs (Table 1). For five of the 33 GCMs in NEX-DCP30 it was not possible to downscale vapor pressure because necessary data from GCMs were not available. As with NEX-DCP30, data are available for RCP4.5 and RCP8.5 climate change scenarios. The data are in annual 12-month files, so that there are 255 files per GCM. The total dataset requires approximately 2.5 TB of storage. Files are named in the following format:

**Table 1:** Global climate models (GCMs) included in this dataset, source institution, and native resolution.

GCM	Institution	Native resolution
ACCESS1.0	The Commonwealth Scientific and Industrial Research Organization (CSIRO) and the Bureau of Meteorology (BoM)	192 × 145
BCC-CSM1.1	Beijing Climate Center	128 × 64
BCC-CSM1.1m	Beijing Climate Center	320 × 160
BNU-ESM	Beijing Climate Center	128 × 64
CanESM2	Canadian Centre for Climate Modelling and Analysis	128 × 64
CCSM4.0	The National Center for Atmospheric Research (NCAR) and the University Corporation for Atmospheric Research (UCAR)	288 × 192
CESM1(BGC)	The National Center for Atmospheric Research (NCAR) and the University Corporation for Atmospheric Research (UCAR)	288 × 192
CESM1(CAM5)	The National Center for Atmospheric Research (NCAR) and the University Corporation for Atmospheric Research (UCAR)	288 × 192
CNRM-CM5	Centre National de Recherches Météorologiques (CNRM) and Centre Européen de Recherche et Formation Avancée en Calcul Scientifique (CERFACS)	256 × 128
CSIRO-Mk3.6.0	Commonwealth Scientific and Industrial Research Organization; Queensland Climate Change Centre of Excellence	192 × 96
FGOALS-g2	Institute of Atmospheric Physics, Chinese Academy of Sciences, and the Earth System Science Research Center at Tsinghua University	128 × 60
FIO‐ESM	First Institute of Oceanography	128 × 64
GFDL-CM3	National Oceanic and Atmospheric Administration; Geophysical Fluid Dynamics Laboratory	144 × 90
GFDL-ESM2G	National Oceanic and Atmospheric Administration; Geophysical Fluid Dynamics Laboratory	144 × 90
GFDL-ESM2M	National Oceanic and Atmospheric Administration; Geophysical Fluid Dynamics Laboratory	144 × 90
GISS-E2-H-CC	National Aeronautics and Space Administration; Goddard Institute for Space Studies	144 × 90
GISS-E2-R	National Aeronautics and Space Administration; Goddard Institute for Space Studies	144 × 90
HadGEM2-AO	Met Office Hadley Centre	192 × 145
HadGEM2-CC	Met Office Hadley Centre	192 × 145
HadGEM2-ES	Met Office Hadley Centre	192 × 145
INM-CM4	Institute of Numerical Mathematics	96 × 96
IPSL-CM5A-LR	Institut Pierre-Simon Laplace	96 × 96
IPSL-CM5A-MR	Institut Pierre-Simon Laplace	144 × 143
IPSL-CM5B-LR	Institut Pierre-Simon Laplace	96 × 96
MIROC5	Atmosphere and Ocean Research Institute; Centre for Climate System Research - National Institute for Environmental Studies; Japan Agency for Marine-Earth Science and Technology	256 × 128
MIROC-ESM	Atmosphere and Ocean Research Institute; Centre for Climate System Research - National Institute for Environmental Studies; Japan Agency for Marine-Earth Science and Technology	128 × 64
MIROC-ESM-CHEM	Atmosphere and Ocean Research Institute; Centre for Climate System Research - National Institute for Environmental Studies; Japan Agency for Marine-Earth Science and Technology	128 × 64
MRI-CGCM3	Meteorological Research Institute	20 × 160
NorESM1-M	Norwegian Climate Center	144 × 96

“BCSD_0.008deg_vpr_” + [GCM] + “_” + [scenario] + “_” + YYYYMM + “-“ + YYYYMM + “.nc”, where GCM refers to one of the 28 global climate models, and scenario refers to the climate change scenario. Because RCP4.5 and RCP8.5 data have the same data for the time period 1950 to 2005, the files representing that period include the phrase “historical”. This is not to be confused with vapor pressure data associated with the PRISM reference climate data [10]. The first set of YYYY and MM represents the beginning year and month, respectively; and the second set represents the ending year and month. For example, the file named

BCSD_0.008deg_vpr_ACCESS1-0_rcp85_210001-210012.nc contains VPR data downscaled from ACCESS1-0 GCM output for January 2100, to December 2100, for the RCP8.5 climate change scenario.

The downscaled vapor pressure dataset is available at the Forest Service Research Data Archive [11]. Vapor pressure values can be readily converted to vapor pressure deficit by subtracting it from saturated vapor pressure, which can be calculated from temperature. There are many empirically derived equations describing the calculation of saturated vapor pressure from temperature [12].

**Fig. 1:**
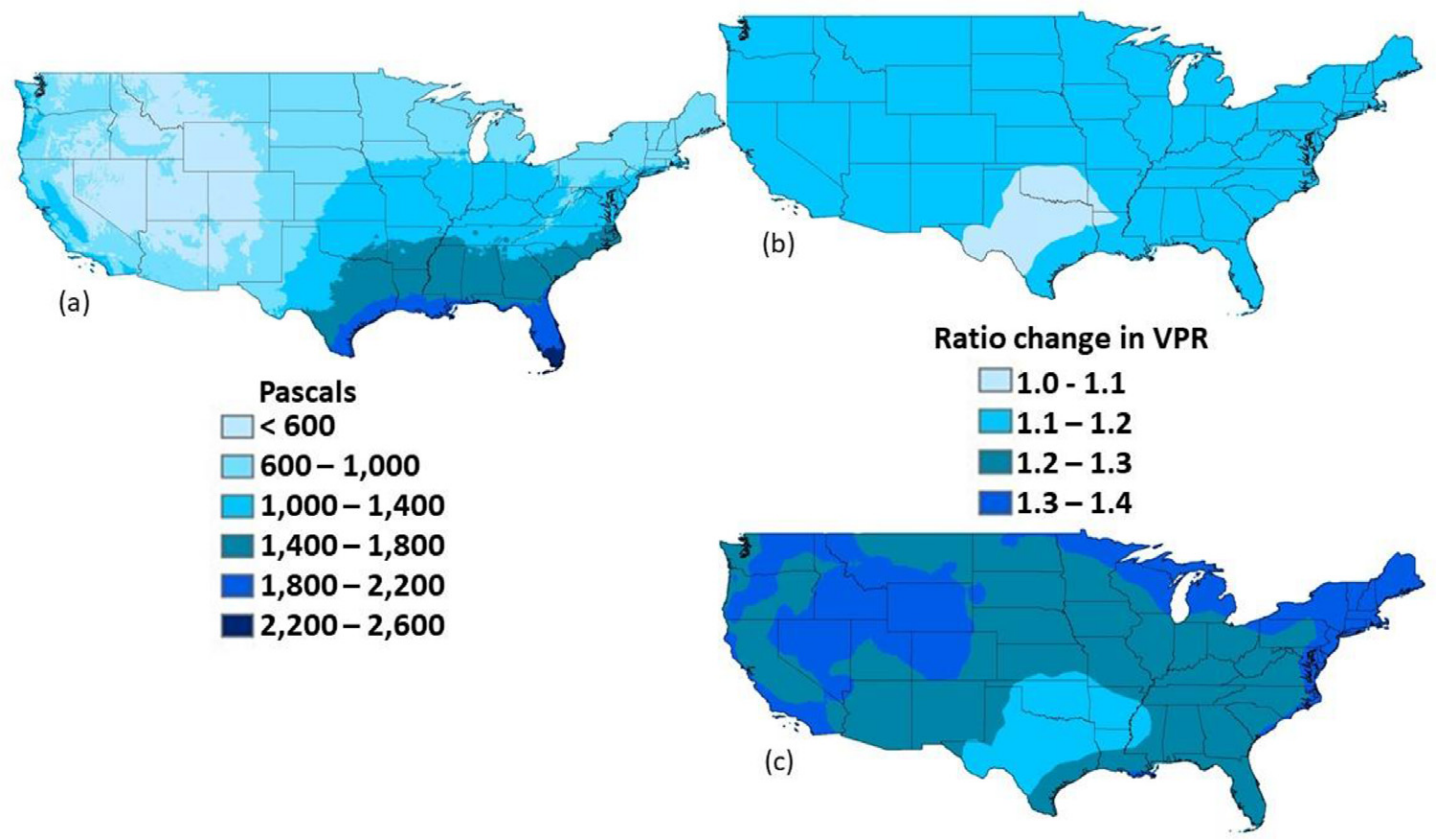
Downscaled vapor pressure for historical and future periods. Average annual vapor pressure (VPR) for the historical period 1976-2005 was calculated from 28 global climate models (GCMs), downscaled to 30 arcseconds (a). The ratio of change for late century (2070-2099) for RCP4.5 (b) and RCP8.5 (c) also represent the average of 28 GCMs, relative to the historical period. Maps are drawn in geographic coordinates, spanning from 24.0625 to 49.9375 in latitude and -125.02083333 to -66.47916667 in longitude.

**Fig. 2:**
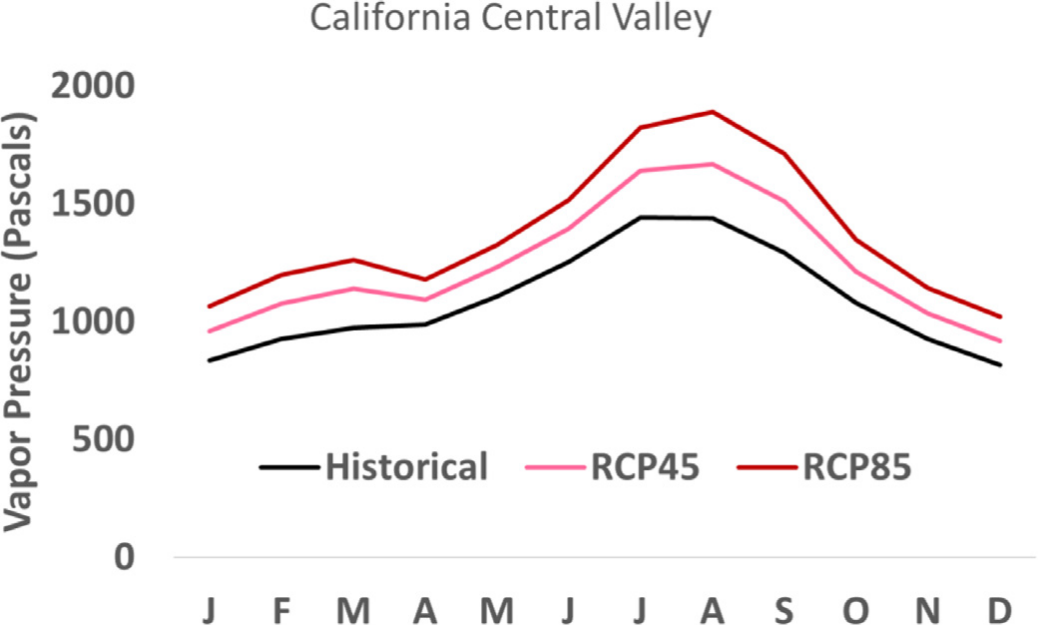
Monthly average vapor pressure values in the Central Valley, CA. Monthly vapor pressure values were averaged from 28 downscaled GCMs for the historical period (1976-2005, black) and the future projected values (2070-2099) for RCP4.5 (pink) and RCP8.5 scenarios (red). Central Valley, CA, is an ecoregion defined in the EPA Level III Ecoregions [13].

Average vapor pressure values were mapped to examine continental scale patterns. Downscaled vapor pressure values exhibit a longitudinal spatial pattern at the continental scale, with the lowest vapor pressure averages in the interior West, and the highest vapor pressure in the Southeast (Fig. 1a). Projected changes into the future are moderate (1.0 - 1.2x historical) under RCP4.5 climate change scenario, and higher (1.2 – 1.4x historical) under RCP8.5 (Fig. 1b, c). Seasonal average vapor pressure time series for a sampling of EPA Level III Ecoregions [13] exhibit reasonable patterns. For example, in the California Central Valley ecoregion, seasonal patterns of vapor pressure remain consistent into the future, with higher values in late summer (Fig. 2).

## 3 Experimental Design, Materials, and Methods

Global climate model (GCM) data in native resolution were downloaded from the Coupled Model Intercomparison Project 5 (CMIP5) data portal on the Lawrence Livermore National Laboratory (LLNL) node of the Earth System Grid Federation (ESGF) [14]. GCM data were downloaded to the NASA High-End Computing Capability (HECC) platform and all subsequent downscaling was performed on the platform. Since vapor pressure (*vpr*) values are not published in the ESGF data portal, *vpr* was calculated in each GCM's native resolution from two published variables, surface specific humidity (*huss*) and surface air pressure (*ps*), using the relationship *vpr* = (*huss* / (*huss* + 0.622)) * *ps*.

Bias correction spatial disaggregation method (BCSD) requires a reference dataset in the target resolution [7]. Parameter-elevation Relationships on Independent Slopes Model (PRISM) [10], version LT71m, was used as the reference dataset. PRISM covers the conterminous USA at 30 arcsecond spatial resolution. Version LT71m spans 1950 to 2005 at a monthly time step, and was selected to best match the temporal span and resolution of the downscaling performed to create the original NEX-DCP30 dataset [6].

BCSD comprises two steps: bias correction and spatial disaggregation [7]. We performed both steps using NCAR Command Language (NCL) [15]. For bias correction, both the reference data and the raw GCM data were resampled to a common 1 degree resolution grid. Then quantile mapping was applied to each cell in the GCM data to correct bias, using PRISM as the reference data. For the spatial disaggregation step, bias corrected monthly ratio anomalies of GCM output are spatially interpolated to the downscaled resolution, and multiplied to mean historical vapor pressure to obtain the downscaled data.

**Fig. 3:**
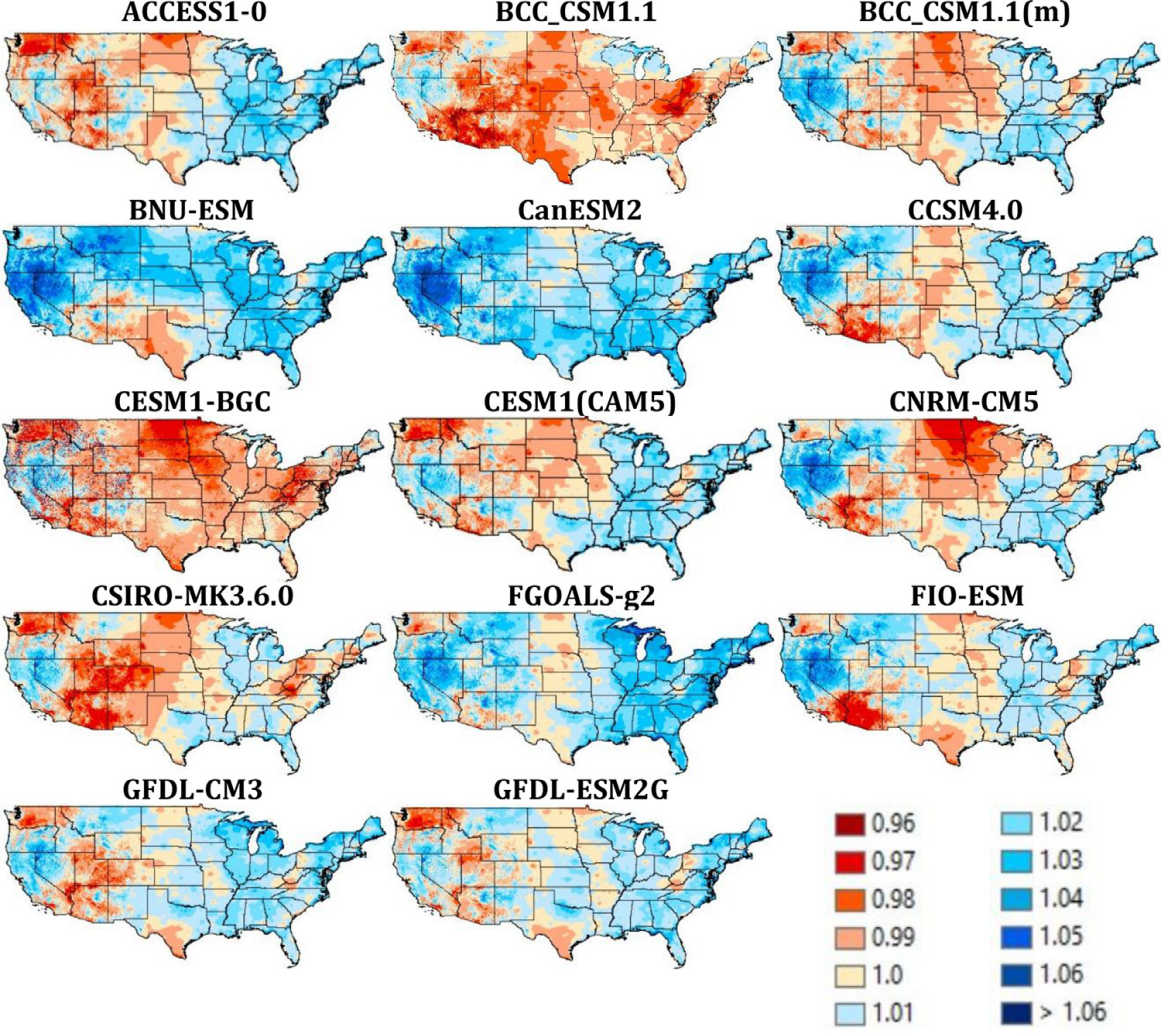

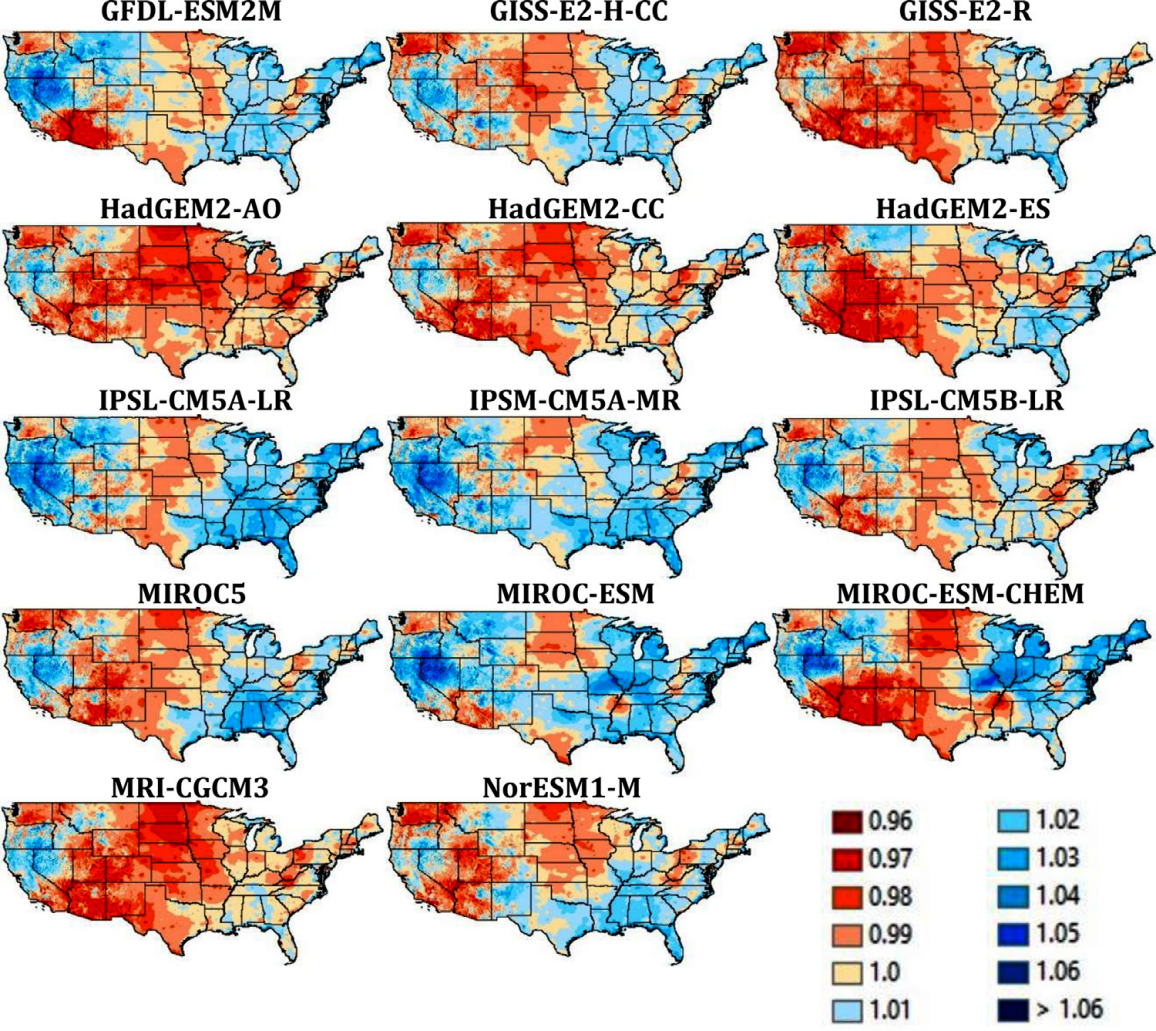
Ratio of downscaled VPR for the years 1980-2009 to reference VPR dataset. The ratios are close to 1, with no outlier ratios.

Quality assurance checks were performed across space and time. First, the average downscaled vapor pressure for the historical period (1980-2009) was compared to corresponding averages derived from the reference data to check for spatial errors (Fig. 3). The maps of the ratio of values, one for each of the 28 GCMs demonstrate that the ratios are close to 1, ranging only from 0.97 to 1.06. Second, the average VPR for each month of the year was plotted for the historical period (1950-2005, Fig. 4) and the future (2006-2099, Fig. 5). No abrupt discontinuities are visible.

**Fig. 4:**
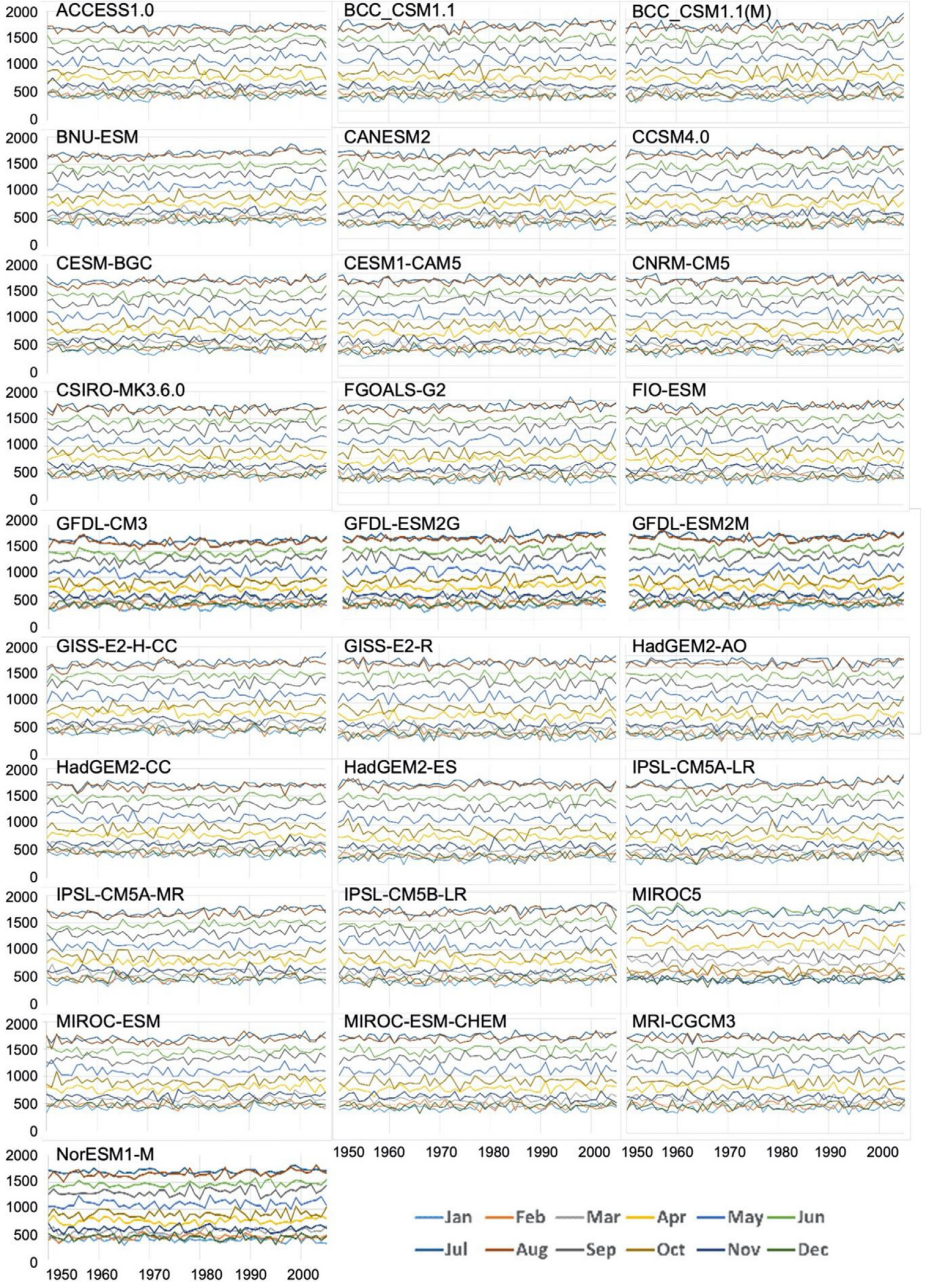
Interannual time series of individual months, 1950-2005. In each panel, each line represents the spatial average of vapor pressure (pascals, y-axis) for the conterminous USA for a month of the year, from 1950 to 2005 (x-axis).

**Fig. 5:**
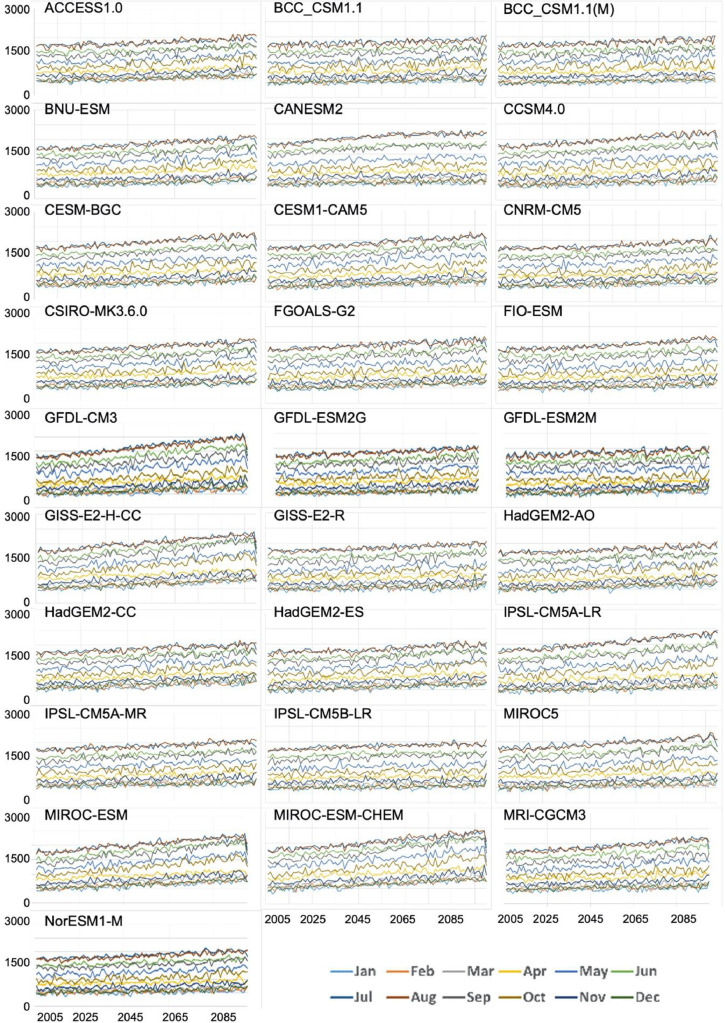
Interannual time series of individual months, 2006-2100. In each panel, each line represents CONUS-wide average vapor pressure (pascals, y-axis) for a month of the year, from 2006 to 2100 (x-axis).

## Ethics Statements

The work in this project did not involve human subjects, animal experiments, or data collected from social media platforms.

## CRediT authorship contribution statement

**Raymond J. Drapek:** Methodology, Software, Validation, Formal analysis, Data curation, Writing – original draft. **Bridget L. Thrasher:** Conceptualization, Methodology, Software. **John B. Kim:** Conceptualization, Methodology, Writing – review & editing, Supervision.

## Declaration of Competing Interest

The authors declare that they have no known competing financial interests or personal relationships that could have appeared to influence the work reported in this paper.

## Data Availability

Dataset of monthly downscaled vapor pressure projections, CMIP5 (Original data) (Forest Service Research Data Archive).

## References

[1] Srivastava L., Hand M., Kim J., Sánchez J.J., Lupi F., Garnache C., Drapek R.J., Quinn J.F. (2020). How will climate change affect the provision and value of water from public lands in Southern California through the 21st century?. Agric. Resour. Econ. Rev..

[2] Halofsky J.E., Bronson J.J., Schaupp Jr W.C., Williams M.P., Kerns B.K., Kuhn B.A., Maxwell C., Kim J.B., Scheller R.M., Halofsky J.E., Peterson D.L., Gravenmier R.A. (2022). Climate Change Vulnerability and Adaptation in Southwest Oregon, Gen. Tech. Rep. PNW-GTR-995.

[3] Kim J.B., Kerns B.K., Drapek R.J., Pitts G.S., Halofsky J.E. (2018). Simulating vegetation response to climate change in the Blue Mountains with MC2 dynamic global vegetation model. Clim. Serv..

[4] Seager R., Hooks A., Williams A.P., Cook B., Nakamura J., Henderson N. (2015). Climatology, variability, and trends in the U.S. vapor pressure deficit, and important fire-related meteorological quantity. J. Appl. Meteorol. Climatol..

[5] Sedano F., Randerson J.T. (2014). Multi-scale influence of vapor pressure deficit on fire ignition and spread in boreal forest ecosystems. Biogeosciences.

[6] Thrasher B., Xiong J., Wang W., Melton F., Michaelis A., Nemani R. (2013). Downscaled climate projections suitable for resource management. EOS.

[7] Wood A.W., Maurer E.P., Kumar A., Lettenmaier D.P. (2002). Long-range experimental hydrologic forecasting for the eastern United States. J. Geophys. Res..

[8] Taylor K.E., Stouffer R.J., Meehl G.A. (2012). An overview of CMIP5 and the experiment design. Bull. Am. Meteorol. Soc..

[9] Moss R., Babiker M., Brinkman S., Calvo E., Carter T., Edmonds J., Elgizouli I., Emori S., Erda L., Hibbard K., Jones R., Kainuma M., Kelleher J., Lamarque J.F., Manning M., Matthews B., Meehl J., Meyer L., Mitchell J., Nakicenovic N., O'Neill B., Pichs R., Riahi K., Rose S., Runci P., Stouffer R., van Vuuren D., Weyant J., Wilbanks T., van Ypersele J.P., Zurek M. (2000).

[10] Daly C., Neilson R.P., Phillips D.L. (1994). A statistical-topographic model for mapping climatological precipitation over mountainous terrain. J. Appl. Meteorol..

[11] Drapek R.J., Kim J.B., Thrasher B.L. (2023). Vapor pressure data for the conterminous United States at a 30 arcsecond resolution for 28 CMIP5 Global Climate Models under RCP 4.5 and RCP 8.5 scenarios. Forest Serv. Res. Data Arch..

[12] Alduchov O.A., Eskridge R.E. (1995). Improved Magnus form approximation of saturation vapor pressure. J. Appl. Meteorol. Climatol..

[13] Omernik J.M., Griffith G.E. (2014). Ecoregions of the conterminous United States: evolution of a hierarchical spatial framework. Environ. Manag..

[14] Cinquini L., Crichton D., Mattmann C., Harney J., Shipman G., Wang F., Ananthakrishnan R., Miller N., Denvil S., Morgan M., Pobre Z. (2014). The Earth System Grid Federation: an open infrastructure for access to distributed geospatial data. Fut.Gener. Comput. Syst..

[15] The NCAR Command Language (Version 6.6.2), UCAR/NCAR/CISL/TDD, 2019. doi:10.5065/D6WD3XH5.

